# Levels of evidence

**DOI:** 10.1093/jhps/hnae047

**Published:** 2025-01-18

**Authors:** Richard E Field

**Affiliations:** City St George’s, University of London, Cranmer Terrace, London SW17 0RE, United Kingdom

One of my mentors once advised me that a journal editor should ask two questions when assessing a manuscript. Firstly, does the paper provide new information? Secondly, does the paper provide evidence to guide the reader’s clinical practice? It was suggested that acceptance for publication should depend on an affirmative answer to both questions. A comprehensive knowledge of the literature and a good search engine should answer the first question. To answer the second question, a judgement must be made on the strength of the evidence provided. In 1979, the Canadian Task Force on the Periodic Health Examination proposed a system to grade the effectiveness of an intervention according to the ‘quality of evidence obtained’ [1]. This led to the concepts of ‘levels of evidence’ and ‘hierarchies of evidence’. The first ‘hierarchy of evidence’ was published by Guyatt and Sackett, in 1995 [2], and the grading outlined by Greenhalgh, in 1997, is familiar to most research clinicians [3]. Over the past 25 years, numerous hierarchies of evidence have been proposed, and in his 2015 PhD thesis [4], Blunt created a database of around 60 published hierarchies. This has recently been updated to include 195 entries [5].

Of the papers published in the *Journal of Hip Preservation Surgery*, systematic reviews and meta-analyses generally secure the most citations. Whether they empower clinicians to develop, evolve, or change practice is less clear. In this issue, a good example of clinical uncertainty is explored in the systematic review from Shane Nho and his team at the Rush University Medical Center in Chicago [6]. From time to time, every hip preservation surgeon will need to advise a symptomatic patient, with borderline hip dysplasia, whether they would be better treated by an arthroscopic intervention or periacetabular osteotomy. While the Rush team has been unable to report on prospective randomized trials comparing the two treatments for comparable patients, they have provided a robust analysis of the available data and this will enable hip preservation surgeons to best provide their patients with realistic expectations for the two treatment options.

In 2004, the opportunity arose to review a paper from Ganz’s team that was subsequently published in, what was, the British Edition of the *Journal of Bone and Joint Surgery* [7]. The paper was not Ganz’s first explanation of femoroacetabular impingement (FAI) [8], but it was probably the paper that provided the wider orthopaedic community a vocabulary to understand the pathological consequences of what they had previously observed as a ‘tilt’ [9] or a ‘pistol-grip’ [10] deformity. The concept of FAI is now well understood and has proven helpful to clinicians and patients alike. Many review articles have been written both from the team who first described FAI [11] and authors from the wider orthopaedic community [12]. While such reviews do not seek to change practice, they do provide a snapshop of current practice among the hip preservation community and can be helpful to surgeons and physical therapists alike. In this issue, we present a review paper from Mexico [13] that will add to this growing body of literature, and we hope that it will be appreciated as a useful overview of the topic.

The lowest level in the hierarchy of evidence are case reports and case series. While there are journals dedicated to publishing case reports [14], most journals are reticent to accept these manuscripts on grounds of limited generalisability, space constraints, perceived lack of novelty, low citation potential, limited resource allocation, and changing perspectives. From time to time, surgeons will seek the advice of their friends on how best to manage an unusual case. Such exchanges can elicit feedback on similar cases, but it is rare for such cases to be pooled for publication as a case series. In this issue, we present a case series from White and his team on their experience of revision labral reconstruction [15]. While it may be some years before such surgery is attempted by the majority of hip preservation surgeons, the paper may prove to be a landmark publication demonstrating what can be achieved in expert hands.

We hope that you find these papers interesting and informative and that they stimulate you to contribute to the growing body of evidence for hip preservation surgery.

## Data Availability

Spitzer W O, Bayne J R D, Charron K C, Fletcher S W, Frappier-Davignon L, Richard R. Goldbloom R R, Morrison B, Offord D, Sackett D L, Gellman D D, Gerry B. Hill G B, Dr. Préfontaine R, Adrian M, Lortie-Monette F, Pless I B, Shephard D A E, Walop W.
